# Flow-Based Dielectrophoretic Biosensor for Detection of Bacteriophage MS2 as a Foodborne Virus Surrogate

**DOI:** 10.3390/bios15060353

**Published:** 2025-06-03

**Authors:** Inae Lee, Heejin So, Kacie K. H. Y. Ho, Yong Li, Soojin Jun

**Affiliations:** 1 Department of Food Science and Biotechnology, Kyung Hee University, Yongin 17104, Republic of Korea; ilee3873@khu.ac.kr; 2 Department of Human Nutrition, Food, and Animal Sciences, University of Hawaii, Honolulu, HI 96822, USA; heejinso@hawaii.edu (H.S.); kacieho@hawaii.edu (K.K.H.Y.H.); liyong@hawaii.edu (Y.L.)

**Keywords:** electrochemical biosensor, foodborne pathogens, microfluidic sensor, carbon nanotubes, dielectrophoresis

## Abstract

Norovirus, a foodborne pathogen, causes a significant economic and health burden globally. Although detection methods exist, they are expensive and non-field deployable. A flow-based dielectrophoretic biosensor was designed for the detection of foodborne pathogenic viruses and was tested using bacteriophage MS2 as a norovirus surrogate. The flow-based MS2 sensor comprises a concentrator and a detector. The concentrator is an interdigitated electrode array designed to impart dielectrophoretic effects to manipulate viral particles toward the detector in a fluidic channel. The detector is made of a silver electrode conjugated with anti-MS2 IgG to allow for antibody–antigen biorecognition events and is supplied with the electrical current for the purpose of measurement. Serially diluted MS2 suspensions were continuously injected into the fluidic channel at 0.1 mL/min. A cyclic voltammogram indicated that current measurements from single-walled carbon nanotube (SWCNT)-coated electrodes increased compared to uncoated electrodes. Additionally, a drop in the current measurements after antibody immobilization and MS2 capture was observed with the developed electrodes. Antibody immobilization at the biorecognition site provided greater current changes with the antibody-MS2 complexes vs. the assays without antibodies. The electric field applied to the fluidic channel at 10 V_pp_ and 1 MHz contributed to an increase in current changes in response to MS2 bound on the detector and was dependent on the MS2 concentrations in the sample. The developed biosensor was able to detect MS2 with a sensitivity of 10^2^ PFU/mL within 15 min. Overall, this work demonstrates a proof of concept for a rapid and field-deployable strategy to detect foodborne pathogens.

## 1. Introduction

Ensuring food safety is a persisting challenge worldwide [1]. Among food safety hazards, harmful bacteria and viruses cause food poisoning in humans via infection or intoxication. According to the US Center for Disease Control and Prevention (CDC), an estimated annual 9.4 million US-acquired foodborne illnesses are due to 31 known pathogens, and most foodborne illnesses are caused by viruses (59%), followed by bacteria (39%) and parasites (2%) [2]. Viruses are considered one of the most infectious pathogens in food industries due to their greater resistance to treatment and the smaller doses required to cause infection. Pathogenic viruses responsible for foodborne illness include norovirus, hepatitis A and E, rotavirus, sapovirus, and astrovirus. In 2016, public health officials from all 50 states in the US reported 875 foodborne disease outbreaks, causing 14,259 illnesses, 875 hospitalizations, and 17 deaths, with norovirus being the leading cause of confirmed, single-etiology outbreaks, accounting for 145 (36%) outbreaks and 3794 (42%) illnesses [3]. Noroviruses are responsible for 58% of viral gastroenteritis, winter diarrhea, and acute non-bacteria gastroenteritis. They are also responsible for approximately 15,000 cases of hospitalizations and 150 deaths in the United States annually [2]. The cost of foodborne norovirus is estimated to be USD 2.3 billion per year due to deaths, non-hospitalized cases, and hospitalizations in the United States [4]. Infection by norovirus is possible even with virus concentrations smaller than 100 copies/mL. Noroviruses are relatively stable in various environments and, thus, it is challenging to prevent them from contaminating water or food. Noroviruses have been reported to survive against up to 10 ppm chlorine, freezing conditions, and temperatures of 60 °C [5]. The potential biological threats to our health and economy emphasize the significance of developing new pathogen monitoring and detection methods.

Pathogenic viruses are transmittable through a variety of routes, including contact with an infected person, contaminated food, water, or surface. Many efforts have been made by food regulatory agencies and manufacturers to minimize the risks of foodborne illnesses, for example, practicing proper hand hygiene, washing and processing fruits, vegetables and shellfish thoroughly, and cleaning contaminated surfaces [6,7]. However, the occurrence of virus-related contamination is still alarmingly prevalent [8]. Currently, there are no regulated detection and identification practices for viral hazards prior to the consumption and use of contaminated water or foods. Most diagnostic tests are only performed after an outbreak has occurred. If norovirus can be preemptively identified, the infection can be blocked from spreading in a public place [9].

Viral detection in a sample has challenges because the viruses are naturally small in size (10–100 nm) and cannot be seen with a standard light microscope. As a result, virus identification usually requires a specific host cell for replication and identification [10]. Current detection or diagnosis methods for viruses are made with the observation of viral particles in solution using a transmission electron microscope (TEM); with the measurement of virus infectivity through the plaque assay and tissue culture infective dose assay (TCID50); or with an assessment of viral protein antigens or gene expressions through hemagglutination assay, single radial immunodiffusion (SRID), enzyme-linked immunosorbent assay (ELISA), and polymerase chain reaction (PCR) [11,12]. Other techniques, such as TEM and the plaque assay, have been widely used as standard methods for determining the quantity of a virus for many decades, but they are time- and labor-intensive and are prone to showing high variability in the results due to operator error. More modern methods based on molecular or serological assays are faster and give more precise and reproducible data than traditional methods [13]. The highest sensitivity for virus detection is achieved with PCR-based assays for the detection of amplified viral DNA and RNA [14]. However, these techniques require multiple pretreatment steps, causing long assay times of up to 24 h, and require specialized equipment and technical expertise, which ultimately make assay-based detection expensive and non-field deployable [15].

Biosensor technologies have been proposed to be a more accurate and fast alternative in viral detection, with less complicated sample preparation steps compared to assay-based techniques [11]. Electrochemical biosensors detect changes via biological recognition events, such as viral antigens binding to specific antibodies placed on the bioreceptor, which can be converted into a quantitative amperometric, potentiometric, or impedimetric signal [10]. These sensors can be used to detect and enumerate intact viruses, viral proteins, and nucleic acids, but the strategy for the direct detection of whole virus particles has advantages of operational simplicity and cost-effectiveness in viral diagnostics [11]. Hong et al. developed a sensitive (35 copies/mL), selective (98%), and rapid (1 h) electrochemical biosensor for the detection of noroviruses. The proposed electrochemical biosensor is designed around a nanostructured gold electrode conjugated with concanavalin A. The oxidation of alkaline phosphatase-labeled secondary antibodies generates a current at the electrode proportional to the amount of norovirus bound to the sensor surface. Another electrochemical immunosensor lab-on-chip immobilized with five types of hepatitis (A, B, C, D, and E) virus antibodies was developed for the simultaneous detection of five-type hepatitis virus antigen with a one-step capture format [16]. The detection is based on the potential change by the antigen and antibody reaction at each detection site. The sensor array can detect most analytes lower than 1.0 ng/mL within 5 min. A label-free electrochemical immunosensor has been shown to detect rotaviruses using gold sononanoparticles immobilized with antibodies [17]. The detection process of the rotavirus involved measurements of electron transfer resistance at the electrode surface, which showed a relationship between measured impedance changes and various rotavirus loads in the range of 4.6 to 4.6 × 10^4^ PFU/mL. A detection limit was determined as 2.3 PFU/mL with a total assay time of 55 min.

Recent advances in microfabrication and nanotechnology have contributed to the miniaturization and automation of biosensor devices with improved sensitivity. Dielectrophoretic (DEP) microdevices have generated a growing interest in bioparticle manipulation and separation over the past decade [18]. Bioparticles, such as DNA, proteins, bacteria, viruses, mammalian, and yeast cells, can travel toward a specific position when subjected to DEP forces. DEP application in the selective analysis of biological samples is possible because particle movement caused by DEP forces is dependent upon the particle structure, morphology, and electrical properties. In addition, DEP forces are further manipulatable by regulating the applied electric field strength, frequency, and electrical conductivity of the suspending medium. The spatial electric field gradients required for DEP effects can be generated by a number of configurations and structures regarding the electrode design and placement within a fluidic channel or sample vessel. Better electric field distribution and control of particle motion can be achieved with modern microfabrication techniques when constructing microelectrode arrays and microfluidic channels [18]. Nanomaterials have begun to play an important role in biosensor design for viral diagnostics. Nanomaterials, such as graphene, carbon nanotubes, quantum dots, and metal nanoparticles, can be used for the isolation and capture of target viral particles from a sample and can also be used to enhance a desired measurement signal [1].

Recently, in vitro cell cultivation systems and animal models have been developed for the detection and separation of infectious norovirus [19,20]. Even though they are promising methods, there are several limitations, in that they are time-consuming and labor-intensive. Due to biosafety issues and resource constraints regarding actual human foodborne viruses, some viral surrogates have been used to model the infectious nature of norovirus in a sample [21,22,23,24]. The F-specific bacteriophage MS2 has frequently been used as a surrogate for human enteric virus studies concerning compounds for disinfecting surfaces in investigating environmental transport and fate [25,26]. Bacteriophage MS2 has similar composition, morphology, size, and site of replication to human norovirus, making it an attractive substitute for food safety studies. Like noroviruses, MS2 is adapted to the intestinal tract, is an icosahedral, positive-sense single-stranded RNA virus, and in the same size range at 26 nm in diameter. Also, it has similar electrical charge and characteristics to norovirus; thus, it is used as a model within virus DEP application studies.

In this study, a flow-based dielectrophoretic biosensor was designed and fabricated as a proof of concept for the rapid and direct detection of norovirus in a sample. Bacteriophage MS2 was used as the norovirus surrogate to evaluate the proposed sensor’s performance. The design and approach are partially based on the author’s doctoral research, which investigated the fundamental principles of DEP for virus detection [27].

## 2. Materials and Methods

### 2.1. Materials

Polydimethylsiloxane (PDMS; Sylgard 184 silicone elastomer curing agent and base) was ordered through Dow Corning (Midland, MI, USA). MG-conductive silver epoxy (# 8331) was purchased from Vetco Electronics (Bellevue, WA, USA). Carboxylic acid-functionalized SWCNTs (SWNT PD1.5L COOH) were manufactured from NanoLab. Inc. (Waltham, MA, USA). Polyethylenimine (PEI, branched, average Mw~25,000), N, N-dimethylformamide (DMF), MES (#M-3671) and bovine serum albumin (BSA; #A3294) were purchased from Sigma Aldrich (St. Louis, MO, USA). 1-ethyl-3-[3-dimethylaminopropyl]carbodiimide hydrochloride (EDC, #22980) and N-hydroxysuccinimide (NHS, # 24500) were supplied from Thermo Fisher Scientific (Waltham, MA, USA). Polyclonal antibody rabbit anti-MS2 IgG was provided by Tetracore Inc. (Gaithersburg, MD, USA). BBLTM tryptic soy broth (TSB), agar powder, and phosphate-buffered saline (PBS) tablets were purchased from VWR (West Chester, PA, USA).

### 2.2. Biosensor Device Fabrication

The device consisted of a PDMS top and bottom structure with a fluid channel and an electrode array, respectively. The molds for PDMS structures were designed using SolidWorks 2020 (Dassault System Solidworks Corp., Waltham, MA, USA) and printed with a 3D printer (Form 2, Formlabs, Somerville, MA, USA) using standard resin. The printed molds were washed several times under an isopropyl alcohol bath and dried at room temperature overnight. The surface of the mold was cured under UV light for one hour. Silicone elastomer curing agent and base were mixed in a ratio of 1:10 and poured into the mold until the channel and electrode guidelines were fully submerged. The molds filled with semi-solid PDMS mixture were placed in the vacuum chamber to remove the bubbles inside and then baked at 65 °C for 1 h. The solidified PDMS layers were peeled off the mold. The negatively printed stations for electrical connections were filled with conductive silver paste. According to the manufacturer’s protocol, the silver epoxy resin and hardener were thoroughly mixed at a 1:1 ratio. The silver mixture was then injected into the electrode channels using a syringe and cured at 65 °C for 20 min.

**Figure 1:**
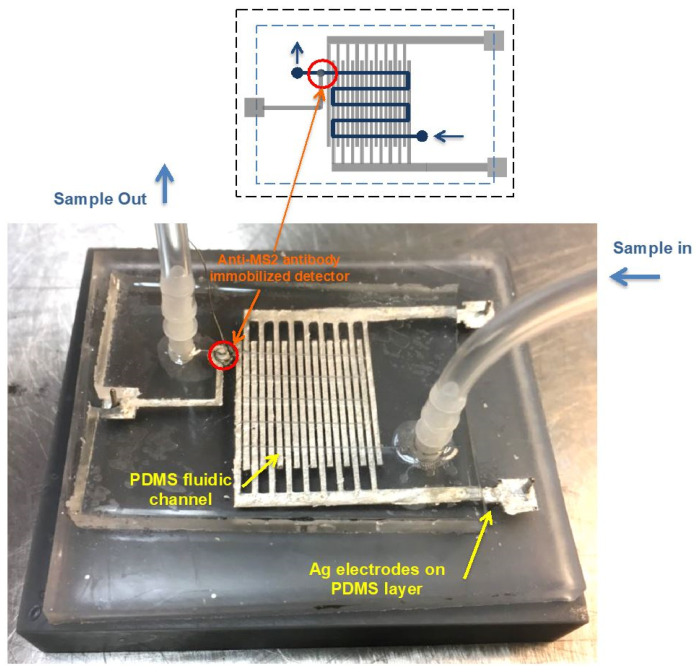
A schematic of the fluidic device, which consists of the PDMS-fluidic channel (Blue), Ag electrode array (Gray) for the DEP generator, and the anti-MS2 lgG immobilized on the SWCNTs coated electrode (Red).The fluid channel, indicated by yellow arrows, crosses the Ag electrode array and is 1 mm in width and 100 μm in height. The width of each strip of the Ag generator electrode strip is 800 μm, with a 400 μm gap between strips. The anti-MS2 IgG immobilized detector located at the end of the channel is marked with orange arrows.

Figure 1 shows a schematic drawing of the fluidic device, which consists of DEP generator and detector electrodes, and a photograph of the fabricated device. There are 80-gap-negative DEP microelectrode arrays exposed within the fluid channel. The electrode width was 800 μm and the gap between adjacent electrode strips was 400 μm. The width and height of the fluid channel were 1 mm and 100 μm, respectively.

### 2.3. Antibody Immobilization on Detector

The detector was placed at the end of the fluid channel and was also filled with conductive silver paste. The anti-MS2 IgG was immobilized on the NHS-ester-activated SWCNTs by covalent linking according to Gomes-Filho et al.’s procedure [28] with slight modification. Then, 10 mg of carboxylic acid-functionalized SWCNTs was dispersed in 5 mL of DMF by sonication under a water bath for 2 h. The SWCNT-COOH suspension was activated with a mixture of 4 mM EDC and 10 mM NHS in 5 mL of 0.1 M MES buffer at room temperature for 1 h. Anti-MS2 IgG and BSA were individually diluted in 10 mM PBS solution (pH 7.2). The detector electrode was first coated with 3 μL of 10% PEI in ethanol and dried at 65 °C for 20 min. NHS-ester-activated SWCNTs (3 μL) were dropped on the PEI film and dried at the same temperature and time. An aliquot of 2 μL anti-MS2 IgG (2.8 mg/mL) was placed on the surface of SWCNT-COO-/PEI/Ag and allowed for peptide coupling between the carboxyl group on SWCNTs and amine group on antibodies at 4 °C for 30 min. Unbound antibodies were washed out with 0.01 M PBS solution. Then, antibody-immobilized electrode was incubated with 3 μL of 2% BSA solution at 4 °C for 4 h to block the non-specific binding sites that are not occupied by anti-MS2 IgG. The two layers were assembled and stored at 4 °C until use and were utilized within two weeks.

### 2.4. Bacteriophage MS2 Propagation

An *E. coli* FAMP strain and MS2 cultures were obtained from Food Microbiology Lab at the University of Hawaii. An inoculant (100 μL) of the frozen stock was transferred to 25 mL of TSB and incubated overnight at 37 °C. The culture amounting to 100 μL was inoculated with another 25 mL of TSB and incubated at the same temperature for 3–4 h until the optical density was between 0.2 and 0.3 at 600 nm, which is the value indicating the logarithmic growth phase. Further, 1 mL of MS2 culture was added to *E. coli* FAMP culture and incubated overnight at 37 °C. The mixture of *E. coli* FAMP and MS2 was centrifuged at 6000 rpm for 10 min, and the supernatant was filtered using a 0.2 μm syringe filter (Corning Inc., Corning, NY, USA) to remove *E. coli* FAMP cells. The bacteria-free MS2 sample was serially diluted in 10 mM PBS.

### 2.5. Bacteriophage MS2 Qualification by Plaque Assay

The viable viral particle counts were determined by the plaque counting method on a double layer of 0.6% and 1.5% tryptic soy agar (TSA). Then, 100 μL of the *E. coli* FAMP cultures, which was in the logarithmic growth phase, was inoculated in the pre-melted 0.6% TSA tube and swirled in the water bath at approximately 50 °C. After adding an equal amount of MS2 sample solution to *E. coli* FAMP-containing TSA tube, they were mixed by rolling in the palm. The mixture was poured out in the pre-warmed 1.5% TSA plates. The plates were incubated at 37 °C for 24 h, and the plaques were counted.

### 2.6. Electrochemical Measurement

Cyclic voltammetry (CV) and electrochemical impedance spectroscopy (EIS) were used for characterization of the functionalized surface on the detector electrode. They were carried out using a μAutolab III/FRA2 (Metrohm Autolab USA Inc., Riverview, FL, USA) controlled by NOVA 1.6 software. The CV experiment was conducted at a potential scan rate of 100 mV/s, step height of 2.4 mV, and applied potential from 1 V to −1 V in an electrolyte solution consisting of 5 mM K_3_Fe(CN)_6_, 5 mM K_4_Fe(CN)_6_, and 0.1 M KCl. In the EIS measurement, the frequency range was from 0.1 to 100 kHz with a DC offset of 200 mV and AC amplitude of 10 mV in the same electrolyte solution. The Nyquist plots were fitted by the built-in analytical tool in the NOVA software, and then electron transfer resistance (R_et_) for the redox reaction at the electrode–film interface was obtained from the Randles equivalent circuit model [R_s_ (C_dl_ [R_et_ Z_W_])], where R_s_ represents the solution resistance, C_dl_ is the double-layer capacitance, R_et_ denotes the electron transfer resistance, and Z_W_ corresponds to the Warburg impedance [29].

### 2.7. Dielectrophoretic MS2 Detection

**Figure 2:**
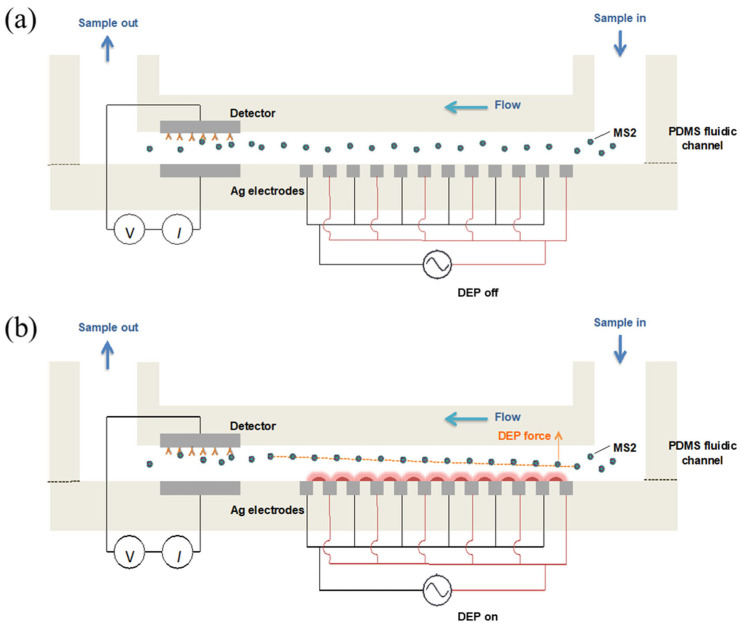
Schematic illustrations for a negative DEP manipulation of MS2 particles to biorecognition site in the fluidic channel. The MS2 particles randomly react with antibodies when no DEP force applied (**a**). MS2 experiences the negative DEP force, which repels from higher electric field gradient, can travel toward the bottom of the fluidic channel and then bind to the antibody immobilized on the detector electrode (**b**).

A syringe pump (Chemyx Inc., Stafford, TX, USA) was used to induce a flow rate of 0.1 mL/mL. A function generator (3220A, Agilent Technologies, Santa Clare, CA, USA) was applied to the interdigitated Ag electrode array to produce the non-uniform electric field in the fluidic channel, with a voltage of 10 V_pp_ and a frequency of 1 MHz. Figure 2 depicts the strategy for MS2 capture on the biorecognition site using a negative DEP manipulation in the fluidic channel. The electrical current signal (*I*) on the detector was measured using a picoammeter (6485, Keithley, Cleveland, OH, USA) with an applied voltage of 0.2 V. The background current measurement (I_antibody_) was conducted with 10 mM PBS. Further, 1 mL of MS2 sample solution was allowed to pass through the detector. By flowing 1 mM PBS into the channel, the unbound MS2 particles washed out from the detector. The current signal after MS2 binding reaction (I_antibody-MS2_) was obtained in 10 mM PBS. The changes in current (Δ*I*) in response to MS2 capture on the detector were calculated as I_antibody-MS2_ − I_antibody._

### 2.8. Statistical Analysis

Data were collected from triplicate experiments reproduced on three separate MS2 dilutions. The mean and standard deviations of Δ*I* were calculated for the serial dilutions of MS2 stock culture. The differences between the means were analyzed based on Duncan’s multiple range tests using a single-factor analysis of variance (ANOVA) in Statistical Analysis Software (SAS version 9.4, SAS Institute Inc., Cary, NC, USA) at a 95% confidence level (*p* ≤ 0.05). The independent sample t-test was conducted using SPSS statistic 20.0 at 95% confidence level to compare the Δ*I* means from the MS2 detection results, with and without anti-MS2-lgG or DEP effect.

## 3. Results and Discussion

### 3.1. Characterization of SWCNT-Antibody-Functionalized Surface and MS2 Detection

**Figure 3:**
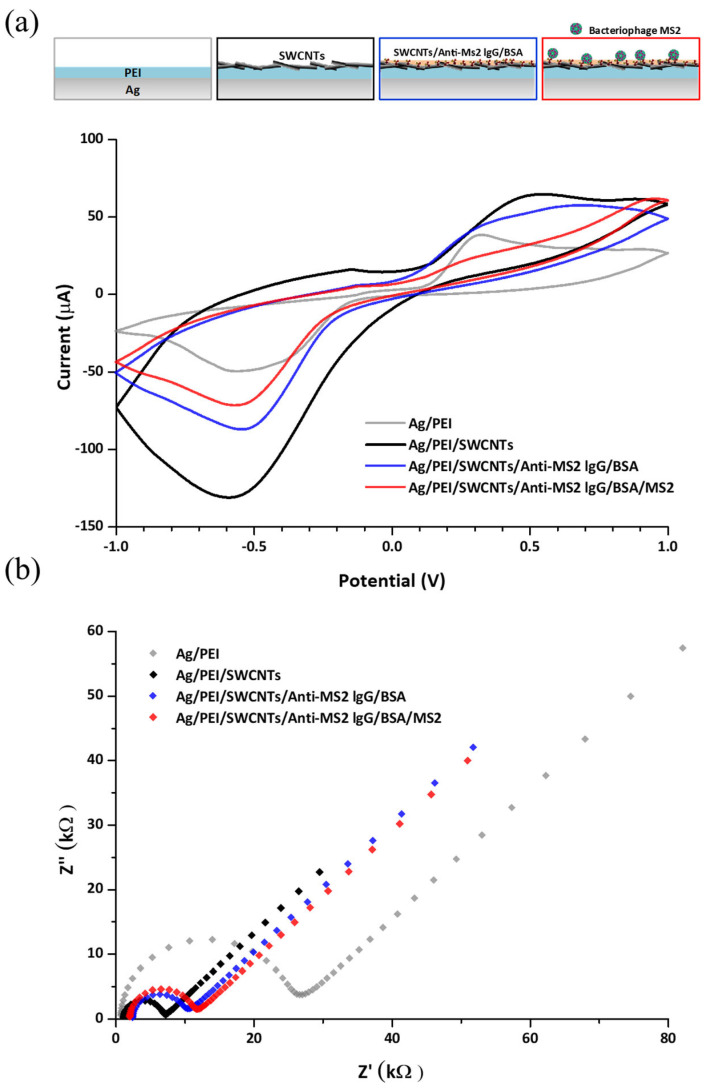
(**a**) Cyclic voltammograms of the detector in each modification step when the potential ranged from 1 V to −1 V at a scan rate of 100 mV/s and (**b**) impedance spectra corresponding to each modification step on the detector electrode in an electrolyte solution consisting of 5 mM K_3_Fe(CN)_6_, 5 mM K_4_Fe(CN)_6_, and 0.1 M KCl.

The surface modification process was characterized by CV and EIS measurements in the presence of the [Fe (CN6)] ^3−/4−^ redox probe. Covalent bonding is one of the conjugation strategies for biological molecules, such as immunoglobulins, to the CNT structure [30]. It has excellent stability and better binding selectivity than non-covalent bonding due to the difficulty in the dissociation of the biomolecules from the nanostructure [31]. The carboxyl groups on the oxidized CNTs are activated by EDC and NHS to form NHS esters, which are highly reactive toward the primary amine groups of antibodies. This reaction results in the formation of stable amide bonds, thereby covalently linking the antibodies to the CNT surface [32]. The first layer of PEI is a highly cationic polymer, which contains a large number of amine groups reacting with the COOH groups of CNTs. Also, the PEI film can offer stable binding of CNTs to the electrode surface. The residues of the COOH group on CNTs can also be linked to the NH_2_ group of the antibody, resulting in the immobilization of antibodies on the electrode. Figure 3a presents cyclic voltammograms of the PEI-coated PEI/SWCNT surface, PEI/SWCNTs/anti-MS2-lgG/BSA-coated Ag electrodes, and MS2 captured electrode. Cyclic voltammograms of the Ag/PEI/SWCNTs electrode showed an increase in the redox peaks in E_pa_ = −0.53 V and E_pc_ = 0.3 V. The voltammetry was decreased in the magnitude of redox peaks after antibody immobilization, followed by MS2 attachment. E_pa_ is the anodic peak potential reached when all of the substrates at the surface of the electrode have been oxidized, and E_pc_ is the cathodic peak potential achieved when all of the substrates at the surface of the electrode have been reduced. The increase in the redox current peaks via the incorporation of CNTs into PEI networks can be explained by the CNTs’ natural high conductivity, which leads to a higher electron transfer to the electrodes, imparting enhanced conductivity at the sensor surface [33]. The addition of biomolecules and MS2 particles contributed to the reduction in the redox peaks due to their insulating properties that can prevent load diffusion to the electrode surface [34].

The electrochemical impedance spectra of the modified electrode are shown in Figure 3b. The diameter of the semicircle indicates that the electron transfer resistance was reduced by SWCNT functionalization and gradually enlarged after antibody immobilization and MS2 attachment to the electrode. This result agrees with the changes in peak current in CV measurement. When the data were applied to Nyquist plots fitted with an equivalent circuit model, the Ag/PEI electrode exhibited a high electron transfer resistance (R_et_) of about 24.0 kΩ. The SWCNT layer affects the reduction in the R_et_ value of the Ag/PEI/SWCNT electrode to 5.04 kΩ, which corresponds to increased redox peaks. As further modifications and MS2 capture progressed, the R_et_ values were increased from 7.30 kΩ for the Ag/PEI/SWCNTs/anti-MS2-lgG/BSA electrode to 8.99 kΩ for the Ag/PEI/SWCNTs/anti-MS2-lgG/BSA/MS2 electrode. Some studies for electrochemical characterization regarding SWCNT-functionalized electrodes showed a similar trend to the results in this study [28,35]. The enhanced current and decreased charge transfer resistance values by CNT modification and shifts in the electrical signals due to the addition of biological molecules were observed.

**Figure 4:**
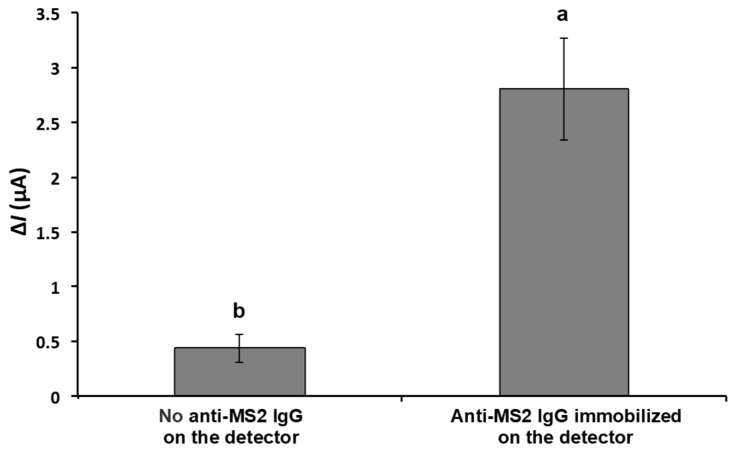
Change in current in response to captured MS2 on the detector with and without MS2 antibody on the surface of detector electrode. The electrical current responses were measured with 10 μL of MS2 solution (~10^10^ PFU/mL) on the detector in stationary mode. Means with different letters are significantly different at 95% confidence level.

Figure 4 indicates the effect of anti-MS2-lgG on the change in the current in response to the MS2 attachment on the detector. The detector electrodes with and without anti-MS2-lgG were incubated with the MS2 droplet (10 μL) containing a concentration of around 10^10^ PFU/mL. The average current change (Δ*I*) increased approximately 6.4-fold from 0.438 ± 0.130 μA (in the absence of anti-MS2-IgG) to 2.806 ± 0.470 μA when the detector electrode was functionalized with anti-MS2-IgG, indicating the successful binding of MS2 particles to the immobilized antibodies.

### 3.2. Effect of DEP Concentration on Change in Signal Response

**Figure 5:**
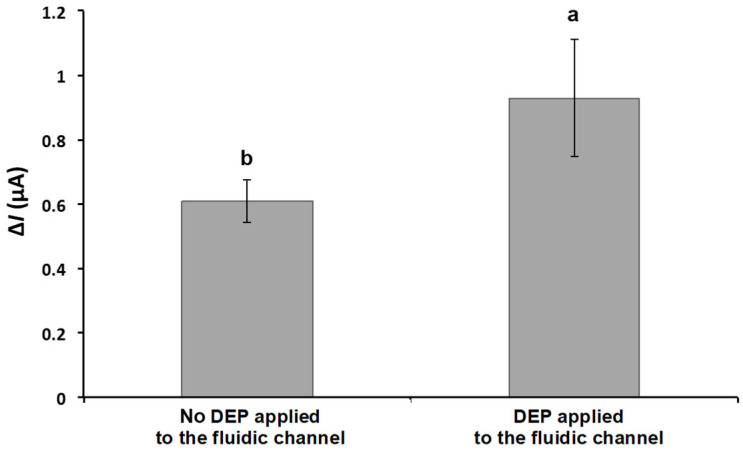
Effect of DEP on current change in response to captured MS2 on the detector. DEP was applied at 10 V_pp_ with a frequency of 1 MHz. The current signals were obtained from the detector filled with PBS after the 1 mL of MS2 solution (~10^7^ PFU/mL) passed through the biorecognition site at the flow rate of 0.1 mL/min. Means with different letters are significantly different at 95% confidence level.

Figure 5 shows the result of Δ*I* with and without the proposed DEP concentration stage applied with an MS2 solution concentration of ~10^7^ PFU/mL. The MS2 solution was introduced into the microfluidic channel at a flow rate of 0.1 mL/min, while an alternating electric field (10 V_pp_, 1 MHz) was applied to induce dielectrophoretic behavior. The average Δ*I* after DEP concentration was 0.930 ± 0.182 μA, which is approximately 50% higher than without DEP applied (0.610 ± 0.067 μA). Hamada et al. studied bacterial detection using both positive and negative DEP [36]. The *E. coli* cells moved toward the impedance detector following negative DEP forces. The peak value of conductance with the nDEP concentration was roughly two-times higher than the values obtained without nDEP concentration.

Similar to bacterial cell manipulation, DEP forces acting on a viral particle depend on the size, shape, conductivity, and permittivity, as well as applied electric field strength, frequency, and suspending medium properties. In this study, a single DEP condition was used at 10 V_pp_ and 1 MHz with 10 mM PBS (a conductivity of 1.5 S/m) as the suspending medium [37,38,39]. However, the control of virus particles due to DEP effect remains a challenge due to their small size [40]. To achieve an enhanced virus concentration, DEP forces and drag forces (i.e., a hydrodynamic force acting upon particles due to flow characteristics and is proportional to the volume of the radius (*r*^3^) and the radius (*r*) of the particle) can be balanced with the proper application of stronger electric fields. Another approach to enhance the virus concentration can be to increase the conductivity of the suspending medium. When the conductivity of the particle is lower than that of the suspending medium, the particle is less polarizable than the medium and experiences negative DEP. Also, the behavior of viral particles can shift depending on the other factors. Therefore, more studies characterizing the DEP effect on virus manipulation are required to better develop biosensor device specificity.

### 3.3. Detection of Bacteriophage MS2 in the Continuous Flow Mode

**Figure 6:**
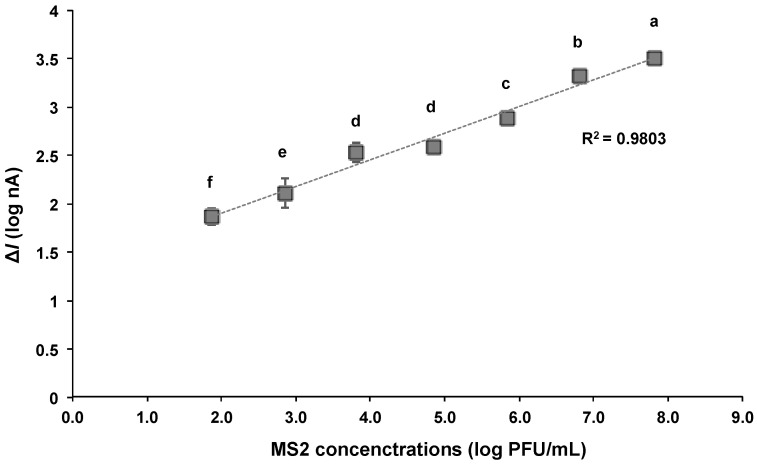
Relationship between logarithmic values of change in current (Δ*I*) and concentrations of MS2 bound to the detector with DEP applied at 10 V_pp_ and 1 MHz. The current was measured at 0.2 V_DC_. Averaged logarithmic Δ*I* values with different letters are significantly different at 95% confidence level.

The electrical current of the PBS solution with various MS2 concentrations ranging from 10^2^ to 10^8^ PFU/mL was measured at a DC potential of 0.2 V. Before conducting the test with the MS2 sample solution, the background current of MS2-free PBS solution was measured. The change in the current of each MS2 concentration compared to the control solution is shown in Figure 6. The Δ*I* value increased with respect to the rising MS2 concentration, and a linear relationship (R^2^ = 0.98) was observed between the logarithmic values of the Δ*I* and MS2 concentration in the range of 10^2^–10^8^ PFU/mL. This implies an increase in the presence of anti-MS2-lgG and MS2 complexes on the detector.

The proposed biosensor in this study is able to provide the detection of MS2 at concentration ranges as low as 100 PFU/mL. In addition, the total assay can be accomplished within 15 min, including particle concentration and current measurement. The bacteriophage MS2 was quantitatively detected in the range of 10^4^ to 10^10^ PFU/mL using reverse-transcription quantitative real-time PCR (RT-qPCR), while the detection limit of the commercial ELISA kit was reported to be 1.2 × 10^7^ PFU/mL [41]. The MS2 detection results based on the biosensor showed comparable or slightly improved sensitivity to these methods (Table 1). The flow-based dielectrophoretic biosensor demonstrated higher detection sensitivity compared to other molecular- or biosensor-based methods, which is presumed to be due to the concentration effect induced by DEP and the microfluidic system. The biosensor integrates a sample pretreatment process that isolates and concentrates trace amounts of target MS2 from small-volume samples, thereby minimizing target loss and enabling highly sensitive detection.

### 3.4. Reproducibility and Stability of the Developed Biosensor

**Figure 7:**
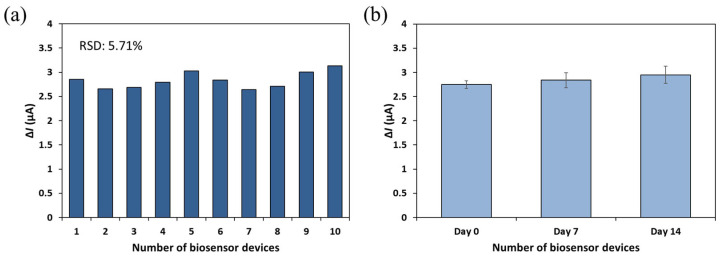
Reproducibility (**a**) and stability (**b**) of the developed biosensor. The current signals were obtained from the detector filled with PBS after the 1 mL of MS2 solution (~10^10^ PFU/mL) passed through the biorecognition site at the flow rate of 0.1 mL/min.

To evaluate the reproducibility of the developed biosensor, ten identical devices were independently fabricated using the same PDMS mold and assembly protocol. The sensor-to-sensor variation in signal output was assessed by detecting bacteriophage MS2 (10^10^ PFU/mL) under the same experimental conditions (Figure 7a). The relative standard deviation (RSD) of the Δ*I* value was calculated to be 5.7%, indicating a good level of reproducibility across independently prepared devices.

For stability assessment, sensors fabricated and subjected to antibody immobilization on the same day were stored at 4 °C under dry conditions and analyzed after one and two weeks (Figure 7b). These sensors were tested using the same concentration of MS2, and the resulting Δ*I* values ranged from 2.748 to 2.949 μA. The calculated RSD was 4.8%, indicating that the biosensor maintained stable performance over the two-week storage period. However, long-term stability and storage performance were not extensively investigated in the current study and will be addressed in future work through the evaluation of sensor performance over extended storage times and under varying environmental conditions.

Overall, the results demonstrate that the developed biosensor exhibits reliable reproducibility and acceptable short-term stability, supporting its potential for practical diagnostic applications.

## 4. Conclusions

In this study, a flow-based dielectrophoretic biosensor was fabricated for the detection of foodborne pathogenic viruses. The newly developed biosensor was evaluated to assess the sensor’s performance when detecting bacteriophage MS2. The incorporation of immobilized antibodies and the SWCNT-modified sensing platform into the biosensor shows great promise for the capture of MS2 via the immunoreaction on the detector electrode. Also, the DEP forces applied to the MS2 suspensions offer potential for virus particle manipulation in the fluidic system. The proposed biosensor was able to detect MS2 in the range of 10^2^–10^8^ PFU/mL (R^2^ = 0.9803), with a total assay time of 15 min. This study demonstrated a proof of concept for the rapid and sensitive detection of viruses using a newly fabricated flow-type sensor. However, the current work is limited in several ways. The DEP conditions used for the viral particle concentration were not systematically optimized, and the mechanical robustness and reusability of the sensor were not evaluated. Future investigations should include selectivity assessments against other foodborne viruses with potential cross-reactivity to establish broader applicability. Additionally, integrating antigen–antibody dissociation and regeneration strategies at the sensor interface may enable sensor reusability. Further efforts should also focus on optimizing DEP parameters to enhance detection sensitivity, as well as establishing appropriate DEP and sensing conditions for application in complex matrices such as real food products and wastewater samples.

## Figures and Tables

**Table 1:** Comparison of MS2 detection sensitivity using biosensor-based methods.

Sensor Type	Detection Limit	Assay Time	Reference
A paramagnetic bead-based electrochemical immunoassay using an interdigitated array electrode in the PDMS fluidic channel	90 ng/mL (1.5 × 10^10^ particles/mL)	-	[42]
An electrochemical bead-based immunoassay in a fluidic system	1.6 × 10^11^ particle/mL	-	[43]
A carbon nanotube-based chemireistive biosensor	10^3^ PFU/mL	5 min	[44]
A porous silicon (pSi) membrane-based electrochemical biosensor	6 PFU/mL	-	[45]
A flow-based dielectrophoretic biosensor	10^2^ PFU/mL	15 min	This study

## Data Availability

The raw data supporting the conclusions of this article will be made available by the authors on request.
